# Supplementary material for: Knowledge, Attitude, and Practice among Physical Therapists toward COVID-19 in the Kingdom of Saudi Arabia—A Cross-Sectional Study
Source: Healthcare (Basel). 2022 Jan 5;10(1):105. doi: 10.3390/healthcare10010105 (PMC8775622; doi:10.3390/healthcare10010105)
Supplement: Supplementary file 1 [file healthcare-10-00105-s001.zip › File-S1.pdf]

healthcare

MDPI

# QUESTIONNAIRE REGARDING KNOWLEDGE, ATTITUDE AND PRACTICE TOWARDS COVID-19

**Kindly reply to these questions either YES or NO**

| KNOWLEDGE | YES/NO |
|---|---|
| **KNOWLEDGE-A**<br>1. COVID-19 is something I've heard about.<br>2. COVID-19 is a potentially contagious virus.<br>3. COVID-19 is caused by which of the following?<br>4. What is the length of the disease's incubation period?<br>5. COVID-19 is treated with which of the following?<br>6. Which age group is more vulnerable to the disease?<br>7. Is Fever being a COVID-19 symptom.<br>8. Is cough a COVID-19 symptom?<br>9. Is a sore throat a COVID-19 symptom?<br>10. Is body discomfort a COVID-19 symptom?<br>11. Is constipation or Diarrhea a sign of COVID-19?<br>12. Is a headache a COVID-19 symptom?<br>13. When I suspect COVID-19 infection, I first check for fever.<br>14. If I suspect COVID-19 infection, the first thing I'll do is see a doctor.<br>15. I shall avoid unnecessary daily activities if I suspect COVID-19 infection.<br>16. To avoid catching COVID-19, I avoid contact with those who are suspected of being contaminated.<br>17. COVID-19 disease is becoming more common in Saudi Arabia.<br>18. Hand washing with soap and water can help to eradicate the disease's source.<br>**KNOWLEDGE-B**<br>19. Coughing is a direct way to spread the sickness.<br>20. Contact with contaminated surfaces can cause the illness to be spread directly.<br>21. Direct transmission of the illness is possible through the ingestion of tainted dairy and meat.<br>22. Direct contact with infected persons (handshakes, hugging, kissing) can transfer the illness.<br>23. Pregnant women are particularly vulnerable to the sickness.<br>24. Older people are more vulnerable to the sickness.<br>25. People with compromised immune systems are more vulnerable to the sickness.<br>26. People with cancer, diabetes, and chronic respiratory disorders are particularly vulnerable to the condition. | |
| **ATTITUDE**<br>1. Early identification of COVID-19, in my opinion, can enhance therapy and result.<br>2. COVID-19, in my opinion, may be handled at home.<br>3. COVID-19 can be prevented, in my opinion, by health education.<br>4. COVID-19, in my perspective, is a serious disease.<br>5. COVID-19, in my view, may be avoided with adequate percussion.<br>6. COVID-19, in my view, may be avoided with adequate percussion.<br>7. COVID-19, in my perspective, is a treatable condition.<br>8. In my perspective, society's knowledge of COVID-19 illness is sufficient.<br>9. COVID-19 sickness, in my opinion, causes death in all cases.<br>10. COVID-19 illness, in my opinion, can be spread to humans through domestic pets.<br>11. To prevent contamination, authorities should restrict travel to and from COVID-19 disease areas, in my opinion. | |

| | |
|---|---|
| 12. Authorities, in my opinion, should confine COVID-19 patients are being treated at specialized hospitals.<br>13. If the number of instances of COVID-19 increases, authorities should be prepared to shut educational establishments, in my opinion (kindergartens, schools, and universities).<br>14. If the number of COVID-19 instances rises, authorities should be prepared to limit access to religious places, shrines, and mosques, in my opinion.<br>15. If the number of COVID-19 cases rises, authorities should be prepared to close and quarantine the city, in my opinion. | |
| **PRACTICE**<br>1. I avoid leaving my house to avoid contracting and spreading COVID-19.<br>2. I avoid taking unneeded vacations to avoid getting and spreading COVID-19.<br>3. I avoid ingesting outside food to avoid catching and spreading COVID-19.<br>4. I avoid shaking hands, embracing, and kissing to avoid contracting and spreading COVID-19.<br>5. I avoid public transportation (taxi, bus, metro, airline, train) to avoid contracting and spreading COVID-19.<br>6. I avoid coming to work to avoid contracting and spreading COVID-19.<br>7. I wash my hands regularly to avoid contracting and spreading COVID-19.<br>8. I devote greater attention to my personal cleanliness than normal to avoid catching and spreading COVID-19.<br>9. I use disinfectants and treatments to avoid contracting and spreading COVID-19.<br>10. I utilize natural items and traditional medicine to avoid catching COVID-19.<br>11. I use vitamin supplements to prevent developing COVID-19.<br>12. When do you apply face masks to avoid contracting and spreading COVID-19? | |
